# Meta-analysis reveals obesity associated gut microbial alteration patterns and reproducible contributors of functional shift

**DOI:** 10.1080/19490976.2024.2304900

**Published:** 2024-01-24

**Authors:** Deep Chanda, Debojyoti De

**Affiliations:** Laboratory of Cellular Differentiation & Metabolic Disorder, Department of Biotechnology, National Institute of Technology, Durgapur, India

**Keywords:** obesity, gut microbial association, meta-analysis, machine learning, functional contributors

## Abstract

The majority of cohort-specific studies associating gut microbiota with obesity are often contradictory; thus, the replicability of the signature remains questionable. Moreover, the species that drive obesity-associated functional shifts and their replicability remain unexplored. Thus, we aimed to address these questions by analyzing gut microbial metagenome sequencing data to develop an in-depth understanding of obese host-gut microbiota interactions using 3329 samples (Obese, *n* = 1494; Control, *n* = 1835) from 17 different countries, including both 16S rRNA gene and metagenomic sequence data. Fecal metagenomic data from diverse geographical locations were curated, profiled, and pooled using a machine learning-based approach to identify robust global signatures of obesity. Furthermore, gut microbial species and pathways were systematically integrated through the genomic content of the species to identify contributors to obesity-associated functional shifts. The community structure of the obese gut microbiome was evaluated, and a reproducible depletion of diversity was observed in the obese compared to the lean gut. From this, we infer that the loss of diversity in the obese gut is responsible for perturbations in the healthy microbial functional repertoire. We identified 25 highly predictive species and 37 pathway associations as signatures of obesity, which were validated with remarkably high accuracy (AUC, Species: 0.85, and pathway: 0.80) with an independent validation dataset. We observed a reduction in short-chain fatty acid (SCFA) producers (several *Alistipes* species, *Odoribacter splanchnicus*, etc.) and depletion of promoters of gut barrier integrity (*Akkermansia muciniphila* and *Bifidobacterium longum*) in obese guts. Our analysis underlines SCFAs and purine/pyrimidine biosynthesis, carbohydrate metabolism pathways in control individuals, and amino acid, enzyme cofactor, and peptidoglycan biosynthesis pathway enrichment in obese individuals. We also mapped the contributors to important obesity-associated functional shifts and observed that these are both dataset-specific and shared across the datasets. In summary, a comprehensive analysis of diverse datasets unveils species specifically contributing to functional shifts and consistent gut microbial patterns associated to obesity.

## Introduction

Obesity, identified as a major global health risk in the 21^st^ century, has nearly tripled since 1975.^1^ Along with genetics^2^ and epigenetics,^3^ a number of other factors, such as demography,^4^ appetite regulation,^5^ lifestyle,^6,7^ and hormone signaling,^5^ play important roles in the etiology of obesity. Recently, independent studies associated the gut microbiome with obesity too.^8–11^ In agreement with these reports, studies related to fecal microbiota transplantation (FMT) being successful in partially reverting obesity-associated host metabolic phenotypes suggest that gut microbial alteration is not merely a consequence but rather may be a cause of obesity-related metabolic imbalance.^12^ Despite these remarkable observations, most of the cohort-specific 16S rRNA sequence-based studies associating gut microbiota with obesity are inconsistent in reporting alteration of microbial community structure such as diversity, Firmicutes/Bacteroides ratio (F/B ratio), etc.^13^ These studies also contradict the finding that differentially abundant taxa are associated with obesity. Moreover, few efforts to generalize the gut microbiome association to obesity by pooling 16S rRNA data from several such studies^9,14^ fail to provide generalizability at the species and functional level because they were based on low-resolution 16S rRNA-based classification, which has inherent problems with sensitivity and reliability.^15,16^ However, pooling high-resolution whole genome sequence (WGS)-based studies with gut microbial species to decipher reproducible pattern associations is lacking. Accordingly, the link between the species and pathway shifts they contribute to, which may provide in-depth insights into the interactions between the host and gut microbiome, remains unexplored. Knowledge of this connectivity is necessary to pinpoint the species contributors to the functional shifts in order to gain a mechanistic understanding as well as to target obesity-associated dysbiosis.

In this study, we present the results of a large-scale meta-analysis of geographically diverse 16S and WGS data aimed at uncovering consistent patterns in the gut microbiota associated with obesity. Our objectives encompassed three primary facets. First, exploring changes in community structure, including shifts in diversity and the F/B ratio. Second, identifying robust taxa and functional signatures and validating them on independent datasets. Third, assessing the influence of potential confounders such as age, sex, comorbidity, and general dietary habits on the patterns linked to obesity. Furthermore, we also sought to establish a link between species and pathways to systematically decipher the drivers of the functional shifts.

## Results

### Search result and sequence dataset selection

**Figure 1.**
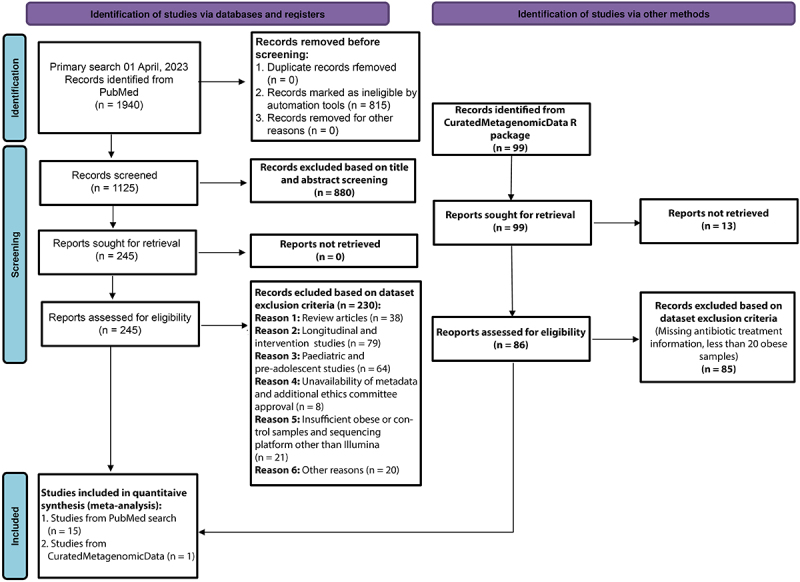
PRISMA flow diagram of the study selection process. Study selection was performed according to the most recent preferred reporting items for systematic reviews and meta-analyses (PRISMA) guidelines.

Pooling data from independent studies improves statistical power due to a larger sample size, and thus can find underlying signals that would otherwise remain unnoticed.^17,18^ Hence, we tried to include as many samples as possible representing different ethnic groups and performed further analyses on the aggregated whole metagenomic datasets, as detailed in the analysis workflow (Figure S1). We searched and selected studies that strictly adhered to the Preferred Reporting Items for Systematic Reviews and Meta-Analyses (PRISMA) guidelines (Figure 1).^19^ Based on the inclusion criteria (see method), we finally included 15 studies with 20 different datasets (10 WGS and 10 16S rRNA gene sequences) in our meta-analysis, which accounted for a total of 3329 samples. WGS datasets comprised 1739 (836 obese, 903 lean control) samples from ten (China, Denmark, Great Britain (GBR), Ireland, Israel, Kazakhstan, Netherlands, Sweden, United Kingdom (UK), and France) different countries, while 16S rRNA gene sequence samples included 1590 (658 obese, 932 control) samples from eight countries (Colombia, Ghana, Jamaica, Poland, Republic of South Africa, Switzerland, United Kingdom (UK), and United States of America (USA)) (Figure S1; Table 1). For our study, we downloaded approximately 12 TB fastq-formatted WGS data and related metadata from the Sequence Read Archive (SRA) using the links provided in the original article (except for the GBR dataset). Additionally, we curated pre-profiled samples that fulfilled the inclusion criteria from a Great Britain cohort (GBR:144 obese and 144 control samples) using the CuratedMetagenomicData R package. The WGS data of the 300-OB cohort were downloaded from the European Genome-Phenome Archive (EGA) with permission from the authors. Due to the absence of a control sample, the 300-OB dataset was only used for training purposes in pooled machine learning analyses, also known as the leave-one-datasetout (LODO) study. Additionally, the French dataset (PRJEB37249) was used for validation. The general characteristics of the datasets and the associated metadata are summarized in Tables 1 and S1, respectively. The 16S rRNA gene sequences were downloaded from SRA.

**Table 1.** Summary of the datasets included in the meta-analysis.

NCBI Bioproject ID	Groups (n)	Age (average ± s.d.)	BMI (average ± s.d.)	Female/male	Country	No. of reads	Assay type	Diet cluster
PRJEB21528 (Jie, Z. et al., 2017)	Control (45) Obese (38)	61.1 ± 9.5	25.9 ± 9.04	53/30	China:1	4.53 × (10^9)	WGS	CHN
PRJEB12123 (Liu, R. et al., 2017)	Control (105) Obese (104)	NA*	NA*	NA*	China:2	10.82 × (10^9)	WGS	
PRJEB1786 (Karlsson, F. et al., 2013)	Control (16) Obese (20)	70.5 ± 0.72	28.9 ± 6.69	36/0	Sweden	1.06 × (10^9)	WGS	SCN
PRJEB1220 (Qin, J. et al., 2010)	Control (55) Obese (99)	56.1 ± 10.32	27.4 ± 6.07	35/33	Denmark	4.53 × (10^9)	WGS	
PRJEB17632 (Kushugulova, A. et al., 2018)	Control (25) Obese (23)	48.7 ± 10.99	27.4 ± 4.89	32/16	Kazakhstan	2.57 × (10^9)	WGS	KAZ
PRJEB11532 (Zeevi, D. et al., 2015)	Control (321) Obese (171)	42.7 ± 12.97	26.42 ± 6.22	305/173	Israel	15.25 × (10^9)	WGS	ISR
PRJEB39223 (Asnicar, F. et al., 2021)	Control (144) Obese (144)	45.2 ± 11.87	27.5 ± 8.20	231/57	Great Britain (GBR)	8.52 × (10^9)	WGS	BIN
PRJEB9576 (Xie, H. et al., 2016)	Control (119) Obese (34)	60.7 ± 8.94	24.86 ± 4.80	153/0	United Kingdom (UK)	10.8 × (10^9)	WGS	
* 300 OB (Kurilshikov, A. et al., 2019)	Control (0) Obese (141)	66.9 ± 5.56	33.3 ± 3.43	64/77	Netherlands	4.85 × (10^9)	WGS	
PRJEB37107 (Ghosh, T. et al., 2020)	Control (39) Obese (39)	76.4 ± 7.98	28.2 ± 6.73	43/35	Ireland	3.32 × (10^9)	WGS	
PRJEB37249 (Independent Validation Set)	Control (34) Obese (23)	46.45 ± 13.36	29.34 ± 9.46	36/21	France	1.21 × (10^9)	WGS	
WGS Total: 1739	Control (903) Obese (836)							
PRJEB6702 (Goodrich, J.K. et al., 2014)	Control (208) Obese (99)	60.91 ± 9.27	26.21 ± 5.91	NA	United Kingdom (UK):1	24.9 × (10^6)	16S	
PRJEB6705 (Goodrich, J.K. et al., 2014)	Control (226) Obese (89)	59.82 ± 9.58	25.55 ± 5.63	NA	United Kingdom (UK):2	23.4 × (10^6)	16S	
PRJEB32880 (Fei, N. et al., 2019)	Control (70) Obese (70)	33.62 ± 5.94	29.61 ± 8.69	89/51	Republic of South Africa (RSA)	2.20 × (10^6)	16S	
PRJEB32880 (Fei, N. et al., 2019)	Control (29) Obese (32)	34.48 ± 6.21	30.54 ± 10.36	47/14	Jamaica	0.95 × (10^6)	16S	
PRJEB32880 (Fei, N. et al., 2019)	Control (129) Obese (25)	35.4 ± 6.8	23.84 ± 5.45	97/57	Ghana	2.61 × (10^6)	16S	
PRJEB32880 (Fei, N. et al., 2019)	Control (32) Obese (102)	36.17 ± 6.04	35.2 ± 9.36	73/61	United States of America (USA):1	1.97 × (10^6)	16S	
PRJNA434133 (Michalovich, D. et al., 2019)	Control (57) Obese (58)	42.48 ± 13.48	NA	79/36	Poland	7.88 × (10^6)	16S	
PRJNA434133 (Michalovich, D. et al., 2019)	Control (19) Obese (21)	41.42 ± 12.28	NA	16/24	Switzerland	10.4 × (10^6)	16S	
PRJNA290926 (Baxter, N.T. et al., 2016)	Control (30) Obese (44)	52.66 ± 9.98	26.87 ± 6.15	48/25	United States of America (USA):2	3.68 × (10^6)	16S	
PRJNA417579 (de la Cuesta-Zuluaga, J. et al., 2018)	Control (132) Obese (118)	40.5 ± 11.22	28.61 ± 6.36	138/112	Colombia	9.91 × (10^6)	16S	
16S Total: 1590	Control (932) Obese (658)							

* 300 OB dataset was used only for training purpose in pooled machine learning experiment, also known as Leave-One-Dataset-Out (LODO).

NA* denotes information not available due to unavailability of metadata.

### Altered species diversity is associated with obese gut with no significant change in F/B ratio

Substantial discrepancies with respect to obesity-associated gut microbial species diversity, gene richness, and F/B ratio have been reported in many cohort-specific studies.^13,20^ To assess whether a pan-ethnic reproducible pattern exists, we initially analyzed these parameters in individual datasets using a univariate analysis. From the univariate non-parametric test on Shannon and Simpson alpha diversity metrics, we found a consistent reduction in gut microbial diversity in obese individuals in majority of the datasets while the reduction was statistically significant in GBR, Israel and Kazakhstan (Figures S2A and S2B). In alignment with the above observations, we also found that obese individuals had reduced gene counts in most of the datasets. The reduction was significant in Denmark, GBR, and Sweden (Figure S2C). However, the opposite trend was observed in the Israel and UK datasets, where the gene count was significantly higher in obese individuals. Furthermore, we could not find any consistent pattern in the F/B ratio except for a significant (*p* =0.00099) increase in China:2 datasets (Figure S2D). We were also interested in determining how the gut microbial composition differed between individuals of a group. For each dataset, we first calculated the compositional dissimilarity (beta diversity) within the control group (“intra-control” beta diversity) and within the obese group (“intra-obese” beta diversity). We subsequently performed the Wilcoxon rank-sum test to assess the differences in beta diversity between the groups. The intra-control beta diversity was higher in the majority of the datasets than in the intra-obese diversity, and it was significant in the Denmark, Ireland, Kazakhstan, and UK datasets (*p* <0.05) (Figure S2E). Next, we calculated beta diversity between the individuals of the obese and control groups as “inter-group” diversity and compared them with “intra-control” diversity as well as with “intra-obese” diversity obtained earlier. As expected, inter-group beta diversity was higher than intra-control and intra-obese beta diversity in the majority of the datasets. Inter-group diversity was significantly (*p* <0.05) higher than intra-obese diversity in Denmark, Ireland, Kazakhstan, Sweden, and the UK datasets (Figure S2E). It was also found to be significantly (*p* <0.05) higher than the intra-control diversity in the China:2, GBR, Israel datasets.

In all these univariate statistical analyses, despite some significant alterations, we observed many inconsistencies across datasets; therefore, we could not deduce any reproducible patterns. Thus, we chose to perform a random-effects meta-analysis to have higher statistical power and accuracy in identifying underlying patterns across datasets.^17^ Pooled analysis of the standardized mean difference (SMD, in terms of Cohen’s *d*) of the alpha diversity indices confirmed its significant reduction across the datasets [for Shannon, meta-analysis coefficient estimate (μ) = 0.20, 95% confidence interval (0.07, 0.33), *p* =0.001) and for Simpson (meta-analysis coefficient estimate (μ) = 0.14, 95% confidence interval (0.01, 0.26), *p* =0.024)] with a very low heterogeneity (Shannon: $I^{2}$ = 21%, *p* =0.36, Q-test; Simpson: $I^{2}$ = 21%, *p* =0.39) (Figure S3A; Table S2). Furthermore, the meta-analysis of gene richness and F/B ratio data revealed no reproducibility in their alterations across the datasets (Figure S3A; Table S2). In agreement with our findings, some reports have questioned the validity of using the F/B ratio as a hallmark of obesity.^14,20^ We further performed a random-effects meta-analysis to assess if a pattern exists with respect to beta diversity alteration across the datasets. Again, we did not find any reproducible association when intra-control and intra-obese beta diversity indices were compared through a meta-analysis. We also probed the reproducibility of the differences in inter-group beta diversity with intra-control and intra-obese beta diversity using a random-effect meta-analysis (Figure S3B). We observed a reproducible pattern with significantly higher inter-group diversity than intra-obese diversity (Figure S3B; Table S2). This observation can be partly explained by the higher compositional variation in the gut microbial community in lean subjects with respect to obese individuals (Figure S3B). Multivariate analyses of reproducible alpha diversity indices using crude and covariate-adjusted coefficients obtained from linear models showed little to no effect of potential covariates (age, sex, and comorbidities) (Figures S3C-S3E).

### Identification of obesity- associated signature taxa

**Figure 2.**
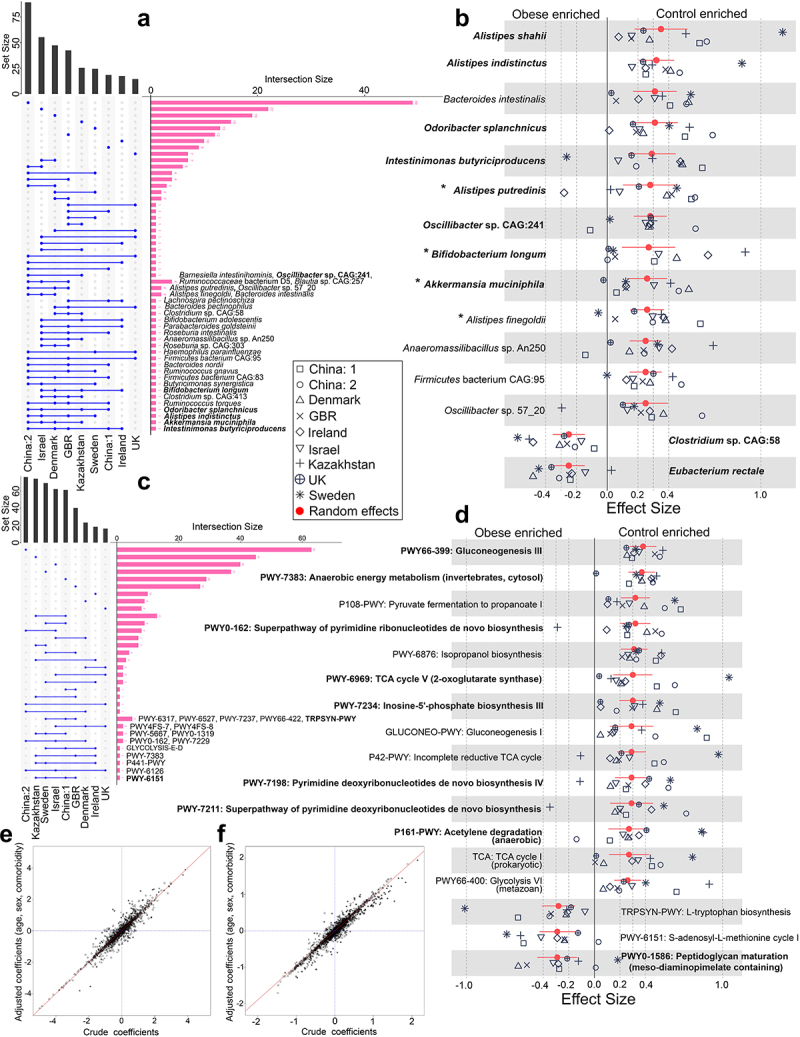
Reproducible taxonomic and functional microbial features across datasets identified by comparing obese and lean controls. (A) The UpSet plot showing the number of reproducible taxonomic features identified using LEfSe on MetaPhlAn3 generated species profiles shared among the datasets. Species highlighted in bold are differentially abundant in at least four datasets and were identified from species-level meta-analysis. GBR, Great Britain; UK, United Kingdom.(B) Pooled effect sizes for the 16 significant species with FDR less than 0.05 and effect size cut off ± 0.25. Red lines represent the 95% confidence interval for the random effects model estimate. Species marked in bold refer to the signatures identified from the machine learning analyses (figure 4). Species marked in ‘*’ denote reproducible contributors of obesity-associated gut microbial signature pathways, as identified from FishTaco analyses (figure 6). (C) UpSet plot showing the number of gut microbial pathway signatures identified using LEfSe on HUMAnN3 generated pathway profiles shared among datasets. Pathways highlighted in bold are differentially abundant in at least four datasets and were identified from the pathway-level meta-analysis. (D) pooled effect sizes for the 17 significant pathways with FDR less than 0.05 and effect size cut off ± 0.25. Red lines represent the 95% confidence interval for the random effects model estimate. Pathways marked in bold refer to the pathway signatures identified from the machine learning analyses. (E-F) Scatter plot of crude and age, sex, and comorbidity adjusted coefficients obtained from linear models using MetaPhlAn3 species abundances (E) and HUMAnN3 pathway abundances (F).

To identify obesity-associated differentially abundant species across the datasets, we initially performed a non-parametric Kruskal-Wallis (KW) test using LEfSe (Linear discriminant analysis Effect Size) tool^21^ on the species relative abundance profiles between the two groups, considering the non-normal distribution of the microbiome composition. We observed that the majority of the species were dataset specific, whereas *Intestinimonas butyriciproducens* found to be lean enriched in at least five datasets. Six other species (*Akkermnansia muciniphila*, *Alistipes indistinctus*, *Odoribacter splanchnicus*, *Ruminococcus torques*, *Clostridium* sp. CAG:413, *Bifidobacterium longum*) were reproducibly differentially abundant in four out of nine datasets (Figure 2a). Except *Ruminococcus torques*, all were lean-enriched. Collectively, our univariate test statistics offer preliminary indications of the presence of consistently observed microbial species associated with obesity across multiple datasets.

To explore reproducibility further, we pooled species-level relative abundance data by random effect meta-analysis, which identified 15 species to be replicable across all the datasets at FDR < 0.05, and effect size cut off ± 0.25 (Figure 2b; Table S3.1). Many of these were also identified in at least four out of nine datasets from the LEfSe analysis (species highlighted in bold in Figure 2a). Among the 13 species that were enriched in the control group, *Alistipes shahii* (μ = 0.35, 95% CI=(0.18, 0.52), *Alistipes indistinctus* (μ = 0.32, 95% CI=(0.20, 0.43), *Bacteroides intestinalis* (μ = 0.31, 95% CI=(0.16, 0.45), and *Odoribacter splanchnicus* (μ = 0.31, 95% CI=(0.16, 0.46) displayed highest effect sizes. However, only two obesity-enriched species were reproducible across datasets: *Clostridium* sp. CAG:58 (μ=-0.25, 95% CI=(−0.35, −0.14) and *Eubacterium rectale* (μ=-0.25, 95% CI=(−0.35, −0.15). We did not find significant heterogeneity across the datasets (Q test, p-value >0.05, $I^{2}$ <60%) (Table S3.1). A similar trend in effect sizes was observed in both Chinese datasets, with some exceptions (*Oscillibacter* sp. CAG:241, *Bifidobacterium longum*, *Anaeromassilibacillus* sp. An250) (Figure 2b). Unlike at the species level, genus-level meta-analysis with 16S rRNA gene sequence data failed to retrieve any strong signals associated with obesity. In fact, at p-value <0.05 and effect size cut off ± 0.25, we did not find any reproducible genera across the datasets. Nonetheless, no effect size cutoff of six significantly (p-value <0.05) reproducible bacterial genera was identified (Figure S4; Table S3.2). Interestingly, we noticed three reproducible genera (highlighted in bold in Figure S4) (*Firmicutes* unclassified, *Alistipes* and *Akkermansia*) whose species were also identified from the species-level meta-analysis, validating the reproducibility of those species. A few observations were specific to the dataset replicates. For example, in the USA dataset replicates in 16S rRNA data (USA:1 and USA:2), *Firmicutes* unclassified, *Clostridium* XIV, and *Akkermansia* showed very similar levels of higher prevalence in the control samples (Figure S4). On the other hand, the *Alistipes*, *Lactobacillus*, and *Roseburia* genera showed opposite trends. Similarly, reproducible genera (*Firmicutes* unclassified, *Alistipes*, and *Akkermansia*) showed similar enrichment in both WGS (Figure 2a) and 16S rRNA meta-analysis (highlighted in bold, Figure S4) in both UK datasets (UK1 and UK2). Nevertheless, not all genera encompassing reproducible species were captured because, in the obese gut, the relative abundance of different species within a genus may exhibit opposite trends in their alterations without significantly affecting the overall relative abundance of that genus. Consistent with prior meta-analyses conducted using 16S rRNA gene sequencing, our analysis^14^ also encountered challenges in accurately capturing the underlying signal. This underscores the importance of employing WGS data to enhance the identification of reproducible patterns.

The results of the species-level meta-analysis were expected to be minimally affected by potential confounders, as indicated by the multivariate analysis of crude and covariate-adjusted coefficients, which were calculated by fitting linear models on species and pathway relative abundances (Figures 2e, f).

### Identification of obesity-associated reproducible pathways

Although univariate non-parametric tests detected reproducibility at the species level, LEfSe analysis at the pathway level revealed that only one gut microbial metabolic pathway (PWY-6151: *S*-adenosyl-L-methionine salvage I pathway) was significantly differentially abundant in at least four datasets (Figure 2c). The rest are either shared by fewer datasets or are exclusively dataset-specific. The outcome of the univariate non-parametric test directed us to further explore reproducibility through random-effect meta-analysis, as it has better statistical power.

Pooled analysis of pathway relative abundance data by random effect meta-analysis retrieved 17 reproducible pathways at FDR < 0.05 and effect size cutoff ±0.25 (Figure 2d; Table S3.3). Of these, 14 pathways were control-enriched and only three were obesity-enriched. Notably, control-enriched pathways were involved in purine and pyrimidine biosynthesis, fermentation, carbohydrate metabolism, and nucleotide sugar biosynthesis (Table S3.3). The enriched pathways with the highest effect sizes (effect size > 0.3) were Gluconeogenesis III (PWY66–399), anaerobic energy metabolism (invertebrates, cytosol) (PWY-7383), pyruvate fermentation to propanoate (P108-PWY), superpathway of pyrimidine ribonucleotides de novo biosynthesis (PWY0–162), isopropanol biosynthesis (PWY-6876), TCA cycle V (2-oxoglutarate:ferredoxin oxidoreductase) (PWY-6969), and inosine-5’-phosphate biosynthesis III (PWY-7234) pathways (Figure 2d; Table S3.3). Our findings support earlier studies that reported a negative association of body mass index (BMI) with nucleotide biosynthesis, glycolysis, gluconeogenesis, and fermentation pathways.^22^ Furthermore, the obese enriched pathways were involved in peptidoglycan maturation (μ=-0.29, 95% CI=(−0.44, −0.13), *S*-adenosyl-L-methionine cycle I (μ=-0.29, 95% CI=(−0.42, −0.13), and tryptophan amino acid biosynthesis (μ=-0.28, 95% CI=(−0.41, −0.15) pathways. Per dataset univariate analyses revealed that the *S*-adenosyl-L-methionine cycle I pathway was significantly enriched in the obese group in four of nine datasets (Kazakhstan, Sweden, China:1, GBR). Thus, although univariate non-parametric analyses failed to find any signature pattern at the functional level, random effects meta-analysis proved effective in identifying underlying patterns.

### Feature-based machine learning model exhibits successful intra-dataset classification but poor transferability across datasets

We further explored the pattern independently using a machine learning-based approach, which is also very powerful for deciphering patterns. We set out to validate whether the signature of independent datasets is transferable to other studies and whether pooling data across studies is more efficient in predicting an underlying pattern. Thus, we performed three sets of prediction tasks using MetAML (Metagenomic prediction Analysis based on Machine Learning)^23^ cross-validation (CV), cross-study validation (CSV), and leave-one-dataset-out (LODO) to check which is the best performing model (see Materials and Methods). First, we performed CV by training and validating the RF (Random Forest) classifier with species relative abundance data on the same dataset and found the AUC (area under the receiver operating characteristic curve) values range between 0.57 and 0.88 with median value 0.67 (s.d. ±0.09) (Figure S5A). We observed training and validation in China 2 dataset resulted in the highest predictive accuracy (AUC = 0.88). A sufficient accuracy level was also achieved when CV was performed on the Denmark (AUC = 0.74), GBR (AUC = 0.73), and Israel (AUC = 0.72) datasets. However, the Ireland and Sweden datasets displayed the lowest classification accuracies. The dataset-specific median accuracy obtained from the CSV ranged between 0.58 and 0.69 (median AUC = 0.60; s.d. ±0.03) (Figure S5A) overall implying a reduced accuracy in CSV in comparison to CV. Since, dataset from different ethnicity had their respective intricacies in features; prediction ability was low when the “training” was done on single dataset followed by validation in different datasets (CSV), possibly due to under fitting.

We also performed similar machine learning-based predictions (CV and CSV) with functional pathway features. We found that the AUC values in CV ranged between 0.50 and 0.79 with a median AUC value 0.68 (s.d. ±0.08) (Figure S5B). In line with the CV at the taxon level, the China:2 dataset displayed the highest accuracy level with an AUC value of 0.79. The Kazakhstan (AUC = 0.75) and Sweden (AUC = 0.71) datasets also attained good classification accuracy. Similar to our observations in taxa, the UK and Ireland datasets displayed the lowest accuracy levels in pathway feature prediction among all the datasets tested, which may be attributed to the inherently low quality of the data and heterogeneity in samples. In addition, when the classifier was trained on one dataset and validated on others (CSV), we observed a reduced median accuracy (similar to taxa prediction exercise) than when it was trained and validated on itself (CV) for all datasets (except Ireland and the UK) (Figure S5B). The dataset-specific median accuracy obtained from the CSV ranged between 0.56 and 0.61 (median AUC = 0.60; s.d. ±0.02) overall implying a reduced accuracy in CSV in comparison to CV as also found with species features.

Altogether, AUC values are comparatively higher in CV than in CSV, suggesting that intra-dataset prediction and validation are specific to datasets and that microbial signatures are poorly transferable across the datasets. This is in line with our dataset specific univariate non-parametric statistical analyses, where differentially abundant species in one dataset were rarely found to be reproducible on other datasets.

### Case versus control discrimination was improved across datasets in LODO setting

The poor study-to-study (CSV) transferability of individual dataset-based models is often attributed to overfitting due to heterogeneity between datasets.^23,24^ This heterogeneity can be attributed to variations in ethnicity, dietary habits, and imbalanced class sizes.^23–25^ To address this issue, we performed LODO and evaluated generalizability of the model. When LODO was performed with species^26^ abundance, as expected, in line with our meta-analysis, we observed a substantially improved accuracy in the transferability of the learning model for all datasets (Figures S5A and S5C). The LODO classification on China:1 and Sweden datasets achieved the best two prediction accuracies (AUC = 0.76) (Figure S5A). A similar level of accuracy was also observed for the Denmark, Kazakhstan (AUC = 0.75), and China:2, GBR (AUC = 0.73) datasets. We also observed improved discriminatory power compared to CV for the UK, Ireland, Sweden, GBR, and Denmark. Overall, we observed a higher median accuracy of LODO (AUC = 0.73) for taxa that exceeded the median accuracy obtained from both CV and CSV experiments. Overall, the inclusion of more datasets in training increased feature diversity, thereby improving the generalizability of the classifier, as we found that this exercise consistently outperformed CV and CSV.

Prompted by the success of the taxa classification, we also performed LODO exercises involving pathway features. In addition, the AUC values improved with respect to the CSV analyses in all datasets, except for outlier Israel (Figures S5B and S5D). The highest accuracy was observed with the Kazakhstan dataset (AUC = 0.75), followed by China:1 (AUC = 0.70), GBR, Ireland, and Sweden (AUC = 0.68) datasets, showing sufficient discrimination performance (Figure S5B). However, if not better, the LODO approach demonstrated an equivalent median prediction accuracy level, achieving an AUC of 0.68, when compared to the cross-validation (CV) method.

Furthermore, the AUC values obtained using the same classifier with shuffled class labels were significantly lower than those obtained from the original class labels with both taxa and functional features (Table S4). Receiver operator curves (ROCs) comparing the original and shuffled class labels in the LODO model revealed improved sensitivity of the model when trained with the original class labels (Figures S5E and S5F). These findings emphasize the specificity of the model for accurately distinguishing between classes.

Finally, we evaluated how the inclusion of feature diversity during the training phase influenced the overall prediction accuracy in LODO. To this end, we computed the AUC values for each validation dataset with a gradually increasing number of training datasets one at a time (Figures S6 and S7) in all possible combinations. At the species-level with LODO exercise, we detected a sharp (0% to 13%; median 11.5%) increase in the AUC values, while including any three training datasets in all possible combinations for majority of the datasets used for validation (China:1, China:2, Denmark, GBR, Kazakhstan, Sweden) (Figures S6A and S6B). Further inclusion of datasets resulted in less prominent changes in the median AUC values. Therefore, the addition of large heterogeneous training datasets promotes an efficient and accurate discrimination between classes. On the other hand, we observed a gradual increase in the AUC value at the pathway level when we included the datasets (3% to 23%; median 11%). We further observed that even with the incorporation of the 9^th^ dataset led to an increase of over 6% in the median AUC value (Figures S7A and S7B). This suggests that there is still potential to enhance the accuracy by including additional datasets, indicating room for improvement in pathway signature detection.

### Obesity associated signature features were deciphered using machine learning based feature ranking

**Figure 3.**
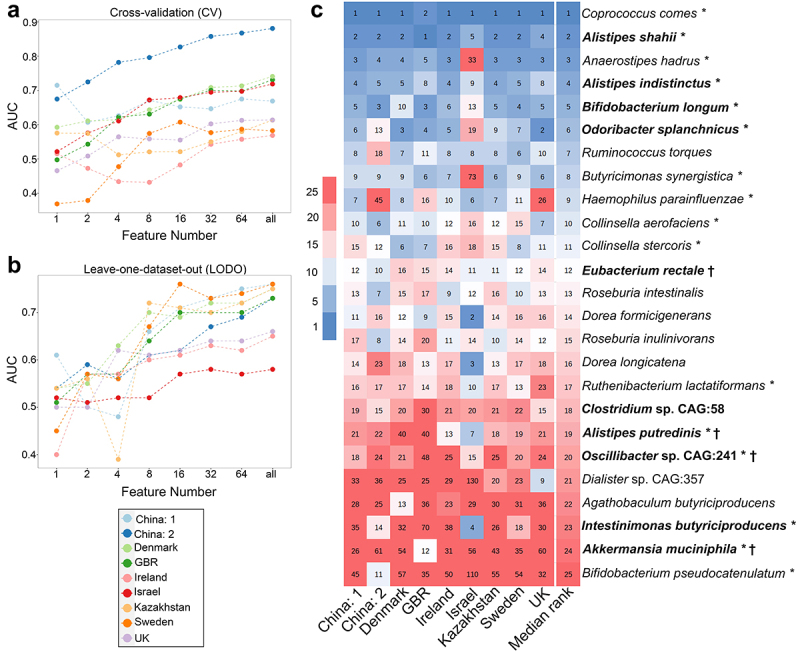
Identification of obesity-associated signature species required for class prediction, their dataset-specific and overall ranking across datasets. (A-B) predictive accuracy with increasing number of species features in cross-validation (CV) (A) and leave-one-dataset-out (LODO) (B) experiment obtained by using backend feature ranking algorithm of random forest classifier. (C) Representation of the rank matrix along with final median rank of top 16 species in each LODO validation making a panel of 25 unique species. Species marked in bold denote signatures also identified from species-level meta-analysis. ‘*’ marked species are the control-enriched signatures as identified from species-level meta-analysis without employing any FDR and effect size cutoffs. Rest is obese enriched. ‘†’ indicates species which were obtained as reproducible contributors of obesity-associated signature pathway shifts from FishTaco (Figure 2 and Figure 6).

**Figure 4.**
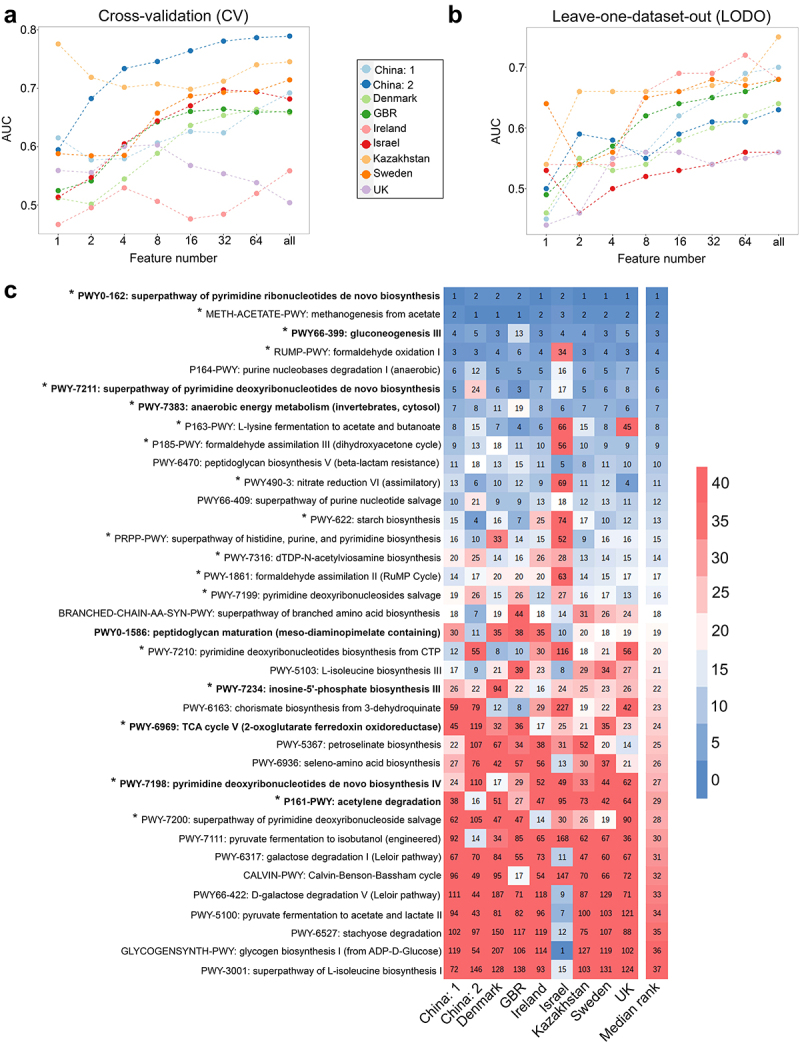
Identification of obesity associated signature pathways required for class prediction, their dataset-specific and overall ranking across datasets. (A-B) predictive accuracy with increasing number of pathway features in cross-validation (CV) (A) and leave-one-dataset-out (LODO) (B) experiment obtained by using backend feature ranking algorithm of random forest classifier. (C) Representation of the rank matrix along with final median rank of top 16 pathways in each LODO validation making a panel of 37 unique pathways. Pathways marked in bold denote signatures also identified from pathway-level meta-analysis. ‘*’ marked pathways are the control-enriched signatures as identified from species-level meta-analysis without employing any FDR and effect size cutoffs.

As LODO mimics meta-analysis and was found to be efficient in classifying samples more efficiently, we used it to identify obesity-associated signature species and pathways (Figures 3 and 4). For this purpose, we looked for a minimum set of features in datasets that can attain an AUC comparable to that obtained using the complete feature set in LODO. While calculating the AUC values with an increasing number of features, we observed that the median AUC value obtained using 16 features did not differ significantly (*p* >0.05) when all features were used for classification in both species (Figures 3a, b) and pathway levels for both CV and LODO settings (Figures 4a, b). This suggests that a finite number of highly predictive species or pathway features can explain the obesity-associated patterns in individual datasets.

To identify signature species associated with obesity, we took 16 top-ranked species from each LODO run and averaged their rankings over all runs, resulting in a panel of 25 species (Figure 3c). These features had the highest discriminatory potential for classifying obese and lean subjects. As expected, many of these species were also found independently through meta-analysis, with 10 out of 15 species found in the said analysis, as highlighted in bold in Figures 2b, 3c, and Table S3.1. Among the top ten ranked species, *Alistipes shahii*, *Alistipes indistinctus*, *Odoribacter splanchnicus*, and *Bifidobacterium longum* were also found to have the highest effect sizes in the meta-analysis.

Subsequently, we identified 16 top-ranked pathways from each LODO run and established a panel of 37 signature pathway features associated with obesity (Figure 4c). We found that 9 out of 17 pathways as deciphered from pathway-level meta-analysis (highlighted in bold in Figures 2d, 4c, and Table S3.3) could also be identified among the top-ranked pathway features obtained from LODO. Several pathways, including PWY66–399, PWY-7383, and PWY0–162, not only ranked highly but also demonstrated among the largest effect sizes in the meta-analysis.

Upon investigating the impact of potential confounders on the identified signature features, we observed that the inclusion of covariates, such as age, sex, and comorbidity (from metadata, Table S1), did not significantly alter the crude estimates obtained from the multivariate analysis (Figures S8A and S9A). This indicates that these covariates did not significantly influence our analysis. This was also confirmed by performing a meta-analysis of the crude- and covariate-adjusted coefficients (Figures S8B and S9B).

### Robust prediction performance by signature features on diet-based clusters

**Figure 5.**
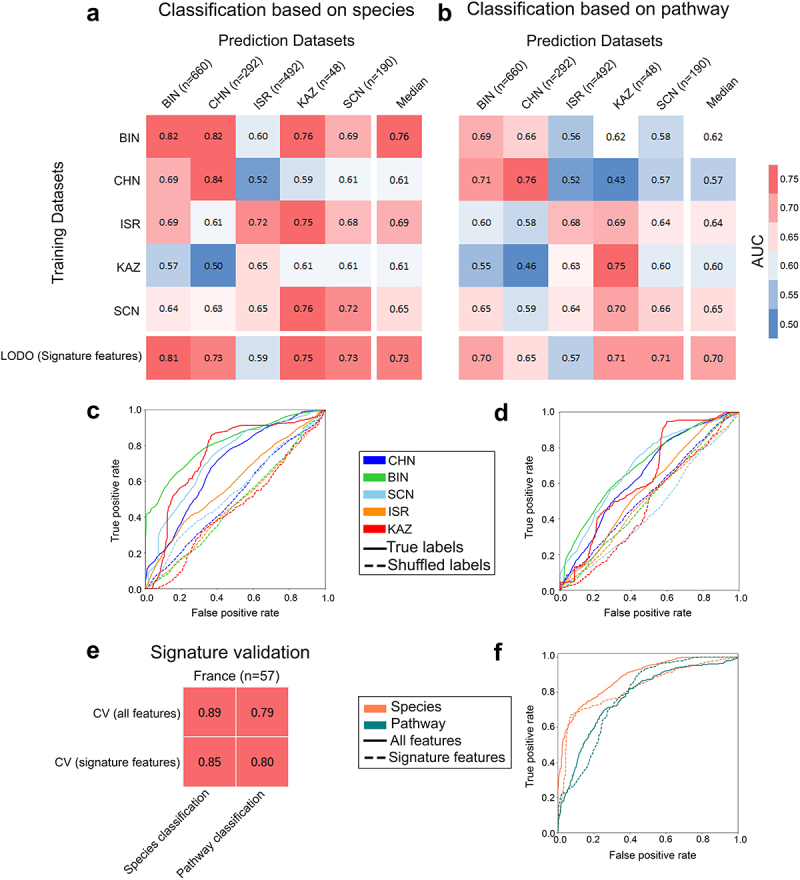
Prediction performance assessment of gut microbiome within and across general diet clusters and validation of signature features. (A-B) Matrix reporting prediction performances in terms of AUC values obtained from MetAML using (A) species and (B) pathway relative abundances. Diagonal values refer to average AUC obtained from 10-fold cross-validations (CV) iterated 20 times. Off-diagonal values report AUC values generated by training classifier on a dataset present in a row and validated on a dataset in the corresponding column representing cross study validation (CSV). Last rows refer to the accuracy achieved by performing LODO with the model generated exclusively using signature features associated to obesity. (C-D) Average ROC curves (over fold) obtained from LODO using (E) species and (F) pathway signature abundance data contrasting sensitivity and specificity of the learning model trained with true and shuffled class labels. (E-F) Prediction accuracy matrix for the models generated from cross-validation (CV) (using all features and signature features) in an independent French validation dataset (E) and the associated ROC curves (over fold) (F) demonstrate utilizing only signature features can yield comparable levels of accuracy to using all features. Diet clusters: BIN (British cluster comprising Great Britain, Ireland, Netherlands, and UK datasets), CHN (Chinese cluster comprising China:1, China:2 datasets); SCN (Scandinavian cluster comprising Sweden and Denmark datasets); ISR (Israeli cluster comprising Israel dataset), and KAZ (Kazakhstan comprising the Kazakhstan dataset).

We next assessed the consistency of the species and pathway signatures on diet clusters (see Materials and Methods) defined by overall dietary patterns using LODO. We observed that using signature features to train and predict resulted in good prediction accuracy (AUC ≥0.70) (Figures 5a & 5b; last row). The results were also evident in the ROCs comparing the LODO learning models independently trained with the true and shuffled class labels (negative control) (Figures 5c,d). The model trained with true class labels exhibited a remarkable enhancement in specificity and sensitivity compared to that trained with shuffled class labels. Signatures performed best for the BIN cluster, followed by KAZ, SCN, and CHN at both the species and pathway levels, but were not very efficient for ISR. We also observed a slight improvement in the median accuracy in CSV (species: AUC = 0.65; pathway: AUC = 0.62) (Figure 5a) following clustering when compared to that without clustering (species: AUC = 0.60; pathway: AUC = 0.60) (Figure S5A). This phenomenon might be the result of reduced overfitting, which was achieved by incorporating diverse data within diet clusters. Additionally, the enhancement of class balance also plays a crucial role in exercise. We further exploited this improvement to identify diet-specific “local” signatures by conducting CV on each cluster and identifying the top discriminatory features required for classification (Figure S10A for taxa and S10B for pathways). We primarily observed that many of the features from the clusters were common to each other and overlapped with global signatures. Some of the high-ranking species like (*Coprococcus comes*, *Anaerostipes hadrus*, *Alistipes putredinis, Alistipes indistinctus*, *Collinsella stercoris*) and pathways like (PWY0–162: superpathway of pyrimidine ribonucleotides de novo biosynthesis) (Figure 3c) were shared among the majority of the clusters (at least in three out of five clusters) and overlapped with the signature features (unclustered) (Figure S10). We also obtained signatures that were exclusively cluster-specific. The specificity of these signatures was restricted to the corresponding clusters from which they were derived and did not populate the top-ranking list obtained from clusters (Figure S10). This phenomenon was further supported by the low CSV values (Figures 5a, b); off-diagonal values). Within the clusters, BIN had the highest overlap of its top 16 taxa features with the signature (Figure S10A), followed by CHN and SCN. Thus, we concluded that despite cluster-specific “local” signatures, incorporation of large, diverse and heterogeneous datasets results in the emergence of more robust “global” signatures (Figures 3c & 4c), which translated into these clusters as well (LODO row in Figures 5a, b).

### Validation of signature features on independent dataset

To further validate our signature features, we obtained an independent metagenomic dataset from France comprising 57 samples (23 obese and 34 lean). We compared the accuracy attained when employing only signature features with respect to the accuracy achieved using all features during classification through CV (Figure 5e). The predictive model utilizing the signature species exhibited impressively high accuracy on the independent dataset, with an AUC value of 0.85. Such high accuracy closely resembles the performance obtained when utilizing all features (AUC = 0.89). Similarly, pathway signatures achieved a high accuracy level (AUC = 0.80), slightly outperforming the accuracy achieved using all pathway features (AUC = 0.79). The ROCs obtained from CV using all features were compared to those obtained from the signature features, and they demonstrated similar levels of sensitivity and specificity (Figure 5f). These observations further strengthened the validity and reliability of the selected species and pathways as robust signatures for classification.

### Reproducible functional shifts are driven by both dataset-specific and reproducible contributor species combinations

We observed a replicable signature pattern at both the taxa and function levels identified by the LODO machine learning experiments (Figures 3b & 3d). However, to understand the contribution of the functional shift associated with obesity, these two feature levels remain to be precisely mapped, which is crucial to gain a glimpse of the biology of obesity and obtain an overview of the therapeutic intervention needed to revert obesity-associated dysbiosis. We attempted to establish this link with the help of FishTaco (*f*unct*i*onal *sh*ifts’ *ta*xonomic *co*ntributors)^27^ metagenomic computational tool (see the method section for details).

Initially, we curated the top 10 direct contributors to the signature pathways (Tables S6.1 and S6.2) for each dataset, and further analyses were performed. For the analyses, we considered the presence of a contributor in at least five out of the nine datasets as the reproducibility criteria. Using this, we identified 16 reproducible contributors (Table S5.3) that drive various pathway shifts (directly or indirectly). The majority of reproducible direct contributors to the control-enriched pathways had a higher prevalence in the control samples (marked with “†”, Table S5.3). Most of these (*Anaerostipes hadrus*, *Alistipes putredinis*, *Akkermansia muciniphila*, *Bifidobacterium longum*) were also identified as signatures (Figure 3c; Table S5.3). Most of the direct contributors of obese-enriched pathways were also identified as signatures (*Eubacterium rectale*, *Ruminococcus torques*, *Dorea longicatena*) (Figure 3c; Table S5.3). This implies that reproducible enrichment or depletion of some bacteria is responsible for obesity-associated functional shifts.

**Figure 6.**
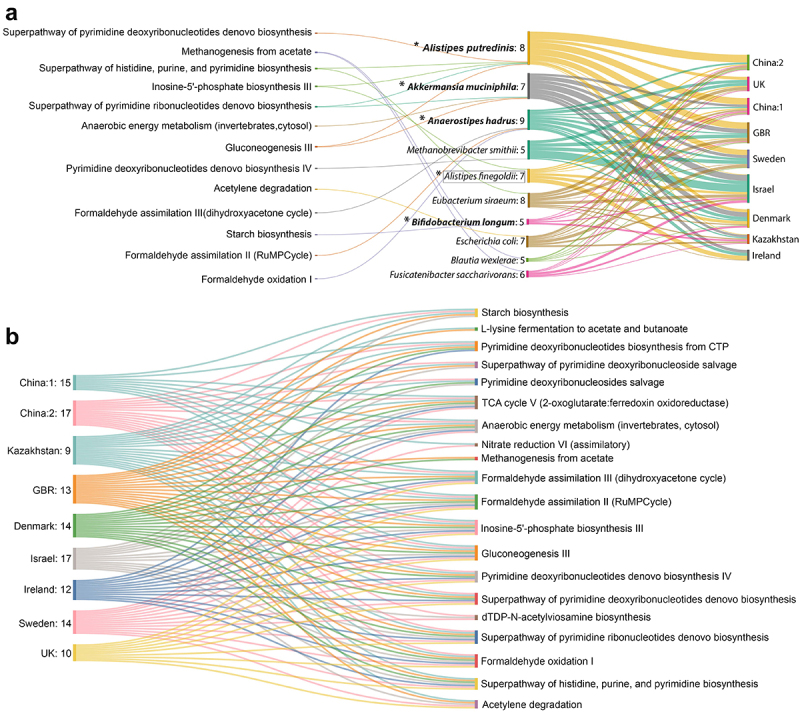
Reproducible contributors of the control-enriched signature pathway shifts and predictive modulation of those pathways by the minimal replicable contributor set. (A) Sankey diagram representing reproducible contributors (5 out of 9 datasets) and their contributed pathways. Values mentioned in second column denotes the number of datasets where a particular contributor drives specific functions it is connected to. Species marked in bold are the ones identified as obesity-associated signatures from MetAML and species bounded with rectangle was identified from meta-analysis. ‘*’ marked are the potential species which were predicted as the minimal set contributing to the maximum number of reproducible pathways. (B) Sankey diagram shows probable control-enriched signature pathways that can be predictively perturbed by minimal reproducible contributor set in different datasets. Values mentioned in each ‘dataset’ column represents the number of control-enriched pathways the minimal set of species directly drives.

Furthermore, we aimed to predict a minimal set of reproducible contributors that may be responsible for partial mitigation of control-enriched pathways in the obese gut microbiome. Thus, we aimed to pinpoint the direct drivers of the control-enriched pathways only (Figure 6a). Five of them (*Alistipes putredinis, Alistipes finegoldii, Akkermansia muciniphila, Anaerostipes hadrus* and *Bifidobacterium longum*) (marked with “*”, Figure 6c) were identified either as signature (in LODO) (marked with “†”, Figure 3c) or retrieved in meta-analysis (marked with “*”, Figure 2b). Hence, these species may have the best potential to directly influence control-enriched pathways across the majority of datasets, thereby aiding in the partial restoration of balance within the dysbiosed gut community. Additionally, we anticipated the pathways in various datasets that could be altered using this limited set of species contributors to partially revive the functional aspects of the community (Figure 6b; Table S5.4). We observed that out of 20 control-enriched signature pathways, the majority of these pathways could be influenced by this specific set of species in almost all datasets.

However, the remaining reproducible contributor species (*Methanobrevibacter smithii, Eubacterium siraeum, Escherichia coli, Blautia wexlerae, Fusicatenibacter sacchanivorans*) were not identified in meta-analyses and machine learning studies. Together with dataset-specific contributors, they collectively drive many pathway shifts (Tables S6.1 and S6.2).

## Discussion

Obesity has been associated with multiple etiologies, including the gut microbiome as one of the important links. Although many previous studies have specifically attempted to demystify this link, there are several unresolved questions related to the reproducibility of community structures, taxonomic and functional features and their association with obesity. This study is an extensive assessment and evaluation of several of these issues where large-scale gut microbial metagenomic sequence data were meta-analyzed from 17 different geographical locations. The choice of studies corresponding to ethnic groups from diverse geographical locations allowed to consider heterogeneities due to variations in geography, host genetics, ethnicity, age, sex, dietary pattern, environment, etc. Thus, this strategy helped to identify obese-associated robust signatures by superseding the bias or noise introduced from batch effects and other confounders.^28,29^ Here, we have reported a consistent reduction in alpha diversity in the obese gut microbiome (Figure S3A), which is also supported by a similar observation in a previous 16S sequence-based meta-analysis.^14^ This observation is relevant in the context of obesity, as it has been rightly concluded that enriched gut microbial diversity is important for resilience and functional performance of the intestinal gut microbial community, which is key to a healthy gut.^30^ In addition, loss of diversity in the context of obesity has been reported and compared to an impoverished ecosystem subjected to fertilizer runoff.^11^ Indeed, a previous report suggested effective resource utilization by diverse gut microbial communities, which in turn promotes a balanced and resilient microbial community capable of imparting health benefits.^31^ Furthermore, high gut microbial diversity maintains functional redundancy in lean individuals, and reduced variation in obesity may lead to a disproportionate increase in selective adverse functions or the loss of beneficial functions. Interestingly, Chatelier et al.^32^ reported a link between reduced gene richness and higher BMI, insulin resistance, increased gut permeability, endotoxemia, and inflammation. However, consistent with a previous 16S rRNA sequence-based meta-analysis,^14^ we also report that F/B ratio does not serve as a robust signature for distinguishing obesity-lean categories (Figure S3A).

In this study, we also established a panel of gut microbial species signatures by pooling datasets using a robust machine learning tool that aligned very well with the findings obtained from our species-level random-effects meta-analysis (Figures 3c, 2b). It is noteworthy that the use of random effects is usually suitable for handling the heterogeneity of data at multiple levels.^33^ The random-effects meta-analysis approach assumes that the studies analyzed in a meta-analysis are not identical; rather, they represent a diverse set of data with varying effect sizes, which is attributed to the inherent heterogeneity within the dataset. This method effectively addresses both within-study heterogeneity arising from random sampling errors and between-study heterogeneity stemming from differences in study characteristics, such as ethnicity and dietary habits. A significant overlap between the random-effects meta-analysis and machine learning-based meta-analysis indicates the reproducibility of the signature features. The validity and robustness of the species and pathway signatures were additionally confirmed using an independent validation dataset from France, demonstrating significantly high accuracy (Figures 5e, f). It is noteworthy that being associated to a different diet cluster France dataset is anticipated to exhibit substantial heterogeneities that are very different from other clusters.^34^ Therefore, the high accuracy of classification with signature features signifies the robustness of the predictive LODO model. Unfortunately, 16S rRNA-based meta-analysis failed to obtain any taxa showing significantly large effect sizes (Figure S4) comparable to meta-analyses from WGS. This problem is primarily attributed to the low resolution inherent in 16S rRNA data.^35^ A previous study performing CV with genus-level abundance data from 16S rRNA also failed to efficiently discern the underlying pattern.^14^ Most of the species established as signatures are control-enriched and have already been established to have beneficial roles in metabolic syndrome or to be negatively associated with high BMI.^22,35–37^ On the other hand, obese-enriched taxa were also linked to impaired host energy metabolism or high BMI.^22^ Functional profiling based on 16S rRNA sequencing was not pursued in this study due to the inherent limitations of 16S rRNA sequencing in providing only a rough estimate of community composition. Among the signature, *Coprococcus comes*, *Alistipes shahii, Alistipes indistinctus, Odoribacter splanchnicus*, *Alistipes putredinis, Oscillibacter* sp. CAG:241, *Intestinimonas butyriciproducens* are short-chain fatty acid (SCFA) producers. SCFAs (such as acetate, butyrate, and propanoate)^38^ are reported to promote energy expenditure, insulin sensitivity, satiety hormone production, and appetite regulation, reduce systemic low-grade inflammation, and thus contribute to improved metabolic health.^39^ Control-enriched signature species may exert their beneficial roles in many different ways. For example, *Anaerostipes hadrus* is believed to contain genes that may facilitate the transport and metabolism of sugar alcohols into butyrate. Butyrate (butanoate), which was enriched in the control group, improved host energy expenditure, insulin sensitivity, and inflammatory regulation, thereby reducing the risk of obesity.^40^ The protective role of butyrate may also be attributed to its epigenetic regulation of host intestinal epithelial cells through reduction of histone deacetylase 3 (HDAC3), as identified in a mice study.^41^ *Akkermansia muciniphila* exerts its protective effect by improving glucose homeostasis, blood lipid levels, and body fat distribution.^42,43^ Likewise, the potential anti-obesity effect of *Odoribacter splanchnicus* and *Ruthenibacterium lactatiformans* could be associated with their anti-inflammatory effect on the gut epithelium, which involves the induction of immune cells to produce interleukin-10 (IL-10) and IFNγ.^44,45^ Additionally, *Akkermansia muciniphila* and *Bifidobacterium* species (*Bifidobacterium longum* and *Bifidobacterium pseudocatenulatum*) have been reported to have beneficial roles by promoting gut barrier function and preventing metabolic endotoxemia due to “leaky” gut.^46^ Remarkably, the study sheds light on certain underexplored bacterial species not previously reported to be associated with obesity in existing literature. These include some control-enriched bacteria like *Butyricimonas synergistica*, *Haemophilus parainfluenza, Collinsella aerofaciens and Collinsella stercoris* which ranked prominently, between 8 and 11 along with obese-enriched *Dialister* sp. CAG:357 (Figure 3).

Similarly, from the metagenomically reconstructed pathways, we obtained statistically significant pathway signatures associated with obesity. The control-enriched pathways mainly belonged to the purine and pyrimidine biosynthetic pathways fermentation pathways, carbohydrate metabolism, and nucleotide sugar biosynthesis pathways (Figure 2d; Table S3.3). Results suggest an enrichment of fermentation pathways that are possibly responsible for producing SCFAs, such as acetate, propionate, and butanoate, as reported earlier.^47^ It is worth mention that enrichment of novel pathways like peptidoglycan biosynthesis and maturation were noticed in the obese group (Figure 4). This finding is in agreement with the enhanced abundance of gram-positive bacteria in the obese group (Figures 2 & 3), as they have higher peptidoglycan content in their cell wall. Peptidoglycan interacts with the host through nucleotide-binding oligomerization domain-containing protein (NOD)-1 and −2 cytosolic receptors, which in turn are responsible for developing insulin resistance and maintaining low-grade chronic inflammation, thus contribute to obesity.^48^ Additionally, some novel control-enriched pathways involved in pyrimidine biosynthesis (PWY0–162, PWY-7211, PRPP-PWY, PWY-7199, PWY-7210), all ranking within top 20, were also identified from this meta-analysis. However, a more detailed mechanistic understanding of these pathways in the context of obesity necessitates further research. Nevertheless, there is potential for further refinement of the pathway signatures through the inclusion of additional datasets (see Figure S7).

The influence of general dietary patterns on obesity-associated signatures were also explored by clustering datasets characterized by similar dietary habits. The slight increase in cross-validation accuracy with clusters (Figure 5) in comparison to the unclustered (Figure S5) underlines the importance of class balance and feature heterogeneity. This exercise also emphasizes the need for cross-cohort studies such as this, which relies on extensive data pooling to establish a generalized pattern, as individual studies are highly dominated by dataset-specific confounders. However, potential confounders such as age, sex, and other reported comorbidities shown to be minimally affect the differentially abundant species and pathways (Figures 2e, f), as well as the signature features (Figure S8A:species, Figure S9A:pathways).

For the first time, in this study, we mapped the microbiome to identify the contributors or the drivers of obesity-associated functional shifts. Contributors were found to be dataset-specific as well as reproducible across datasets (Tables S6.1 and S6.2). As expected, some of the reproducible contributors to function were also found to be obese-associated signatures, as established in the meta-analysis and machine learning experiments (Figure 3c; Table S5.3). Additionally, we noticed that other reproducible contributors together with dataset-specific contributors, despite their insignificant alteration in abundance, drive obesity-associated gut microbial pathways (Tables S6.1 and S6.2). Consequently, it can be inferred from the study that while a consistent set of functional alterations is evident across datasets, these changes may not necessarily be driven by the same set of taxa. Thus, mere identification of differentially abundant species and looking into their functional potential is not enough to understand the biology and strategizing therapy. Therefore, it is crucial to consider intricate interconnectivity within the gut microbial ecosystem.

However, there are certain limitations associated with this study. While efforts were made to minimize gut microbial variations caused by differences in sequencing platforms and comorbidity factors, several other factors could limit the power of this study. These include host genetics and other relevant metadata that are inaccessible to us. In contrast to the species signature, it is evident that further refinement of the pathway signature may be necessary by incorporating additional datasets. A solution could be to analyze and compare matched data (based on different confounders) in case and control samples from different datasets. This requires defining and assessing various confounders associated with these studies. Nevertheless, including a machine learning-based pattern identification that considers all the inherent heterogeneity of the data solves the issue to some extent. In addition, in the context of our study, intestinal mucosa-associated microbiome and virome data, which are currently unavailable, can further enrich the understanding of microbiome association with obesity. An additional constraint of the present study lies in its reliance on direct analyses of microbial abundance in isolation, which overlooks the aspect of microbial interdependencies. Therefore, there is a compelling need for co-abundance network-based analyses that can specifically identify the keystone species responsible for maintaining gut microbial diversity in healthy lean individuals. Establishing a comprehensive framework of microbe-microbe and host-microbe metabolite cross talk in the context of healthy and obese gut environment is essential for gaining a mechanistic understanding of how the loss of diversity contributes to obesity.

To conclude, we have systematically deciphered gut microbiome patterns associated to obesity using large-scale heterogeneous datasets. The depleted gut microbial diversity resulted in the loss of many commensal bacteria and essential functions that could be involved in pathophysiological manifestation of obesity. Panels of taxa and functional signatures of obesity were identified utilizing a machine learning model trained in heterogeneous datasets belonging to diverse ethnic origin. General dietary patterns were often considered as contributing factors involved in modulating gut microbial ecosystem. We observed that taxonomical markers associated to diet-clusters were very similar to the signatures obtained from individual studies, which further underlines the robustness of pipeline used in this report to identify signature pattern. Again, mapping species to functional shifts provides a mechanistic understanding of the microbial contribution to obesity and establishes the foundation for designing therapeutic strategies. However, to further enhance the precision and accuracy of our findings, the incorporation of more datasets might be helpful.

## Materials and methods

### Search strategy and inclusion criteria for public metagenomic sequence dataset selection

Due to the unavailability of a whole genome sequence (WGS)-based dedicated study on obesity, we considered the curation of samples from general cohort-specific studies as well as from case-control studies of other diseases. Thus, we conducted thorough keyword searches in PubMed to retrieve studies with publicly available human fecal shotgun metagenomic data from inception until March 2023. We entered the following fields (((“human s”[All Fields] OR “humans”[MeSH Terms] OR “humans”[All Fields] OR “human”[All Fields]) AND “metagenome/genetics”[MeSH Terms] AND “obesity/genetics”[MeSH Terms]) OR ((“gastrointestinal microbiome”[MeSH Terms] OR (“gastrointestinal”[All Fields] AND “microbiome”[All Fields]) OR “gastrointestinal microbiome”[All Fields]) AND “algorithms”[MeSH Terms]) OR (“obesity/microbiology”[MeSH Terms] AND “gastrointestinal microbiome/immunology”[MeSH Terms] AND “feces/microbiology”[MeSH Terms]) OR (“cardiovascular diseases/microbiology”[MeSH Terms] AND “metabolic diseases/microbiology”[MeSH Terms]) OR (“obesity/microbiology”[MeSH Terms] AND “feces/microbiology”[MeSH Terms]) OR (“gastrointestinal microbiome/physiology”[MeSH Terms] AND “obesity/epidemiology”[MeSH Terms]) OR (“feces/microbiology”[MeSH Terms] AND “colonic diseases/diagnosis”[MeSH Terms] AND “colonic diseases/etiology”[MeSH Terms]) OR (“gastrointestinal tract/microbiology”[MeSH Terms] AND “diabetes mellitus, type 2/microbiology”[MeSH Terms]) OR ((“obes surg”[Journal] OR (“obesity”[All Fields] AND “surgery”[All Fields]) OR “obesity surgery”[All Fields]) AND “obesity/microbiology”[MeSH Terms] AND “obesity/metabolism”[MeSH Terms]) OR (“gastrointestinal microbiome/genetics”[MeSH Terms] AND (“microbiota”[MeSH Terms] OR “microbiota”[All Fields] OR “microbiotas”[All Fields] OR “microbiota s”[All Fields] OR “microbiotae”[All Fields]) AND “human genetics”[MeSH Terms]) OR (“atherosclerosis/microbiology”[MeSH Terms] AND “inflammation/microbiology”[MeSH Terms] AND “liver cirrhosis/microbiology”[MeSH Terms]) OR (“metagenomics/methods”[MeSH Terms] AND (“computational biology”[MeSH Terms] OR (“computational”[All Fields] AND “biology”[All Fields]) OR “computational biology”[All Fields]) AND (“metagenome”[MeSH Terms] OR “metagenome”[All Fields] OR “metagenomes”[All Fields] OR “metagenomic” [All Fields] OR “metagenomically”[All Fields] OR “metagenomics”[MeSH Terms] OR “metagenomics”[All Fields]) AND “microbiota”[MeSH Terms])) AND (1000/1/1:2023/3/31[pdat]).

Additionally, we used the CuratedMetagenomicData R package to include more samples in our study (and hence more statistical power) from pre-profiled fecal metagenome sequences and associated metadata.^49^ For all the datasets considered in our study, we included samples in our meta-analysis if the subjects (1) were adults, (2) met the World Health Organization (WHO) body mass index (BMI) criteria of obese and lean, (3) did not have any gastrointestinal disease, and (4) did not consider a study if the subjects were exposed to antibiotic usage. To reduce bias due to technical variation, we included fecal shotgun metagenome sequenced with the Illumina sequencing platform only. We also ensured that the samples included in our study had important metadata information, such as “Subject ID”, “SampleID”, “Age”, “Sex”, “BMI”, “Country”, “Sequencing platform”, “Total number of reads”, and “Average read length”. In the case of incomplete metadata, we personally communicated the corresponding author(s) to obtain the missing information. Studies requiring additional ethics committee approvals or authorizations or fewer than 20 case samples were excluded from our analysis. The entire schema of the study selection and data retrieval is summarized in Figure 1. Samples with BMI ≥ 18.5 to < 25 without any comorbidity information were considered as “control” samples, while BMI ≥ 30 was regarded as “obese” samples. The BMI criteria were slightly different for Asia-Pacific populations, where samples with BMI ≥ 18.5 to < 23 and BMI ≥ 25 were considered as “control” and “obese” respectively.^50^ We selected another shotgun metagenomic sequence dataset from France, which was used to validate our machine learning model after removing samples with any comorbidity.

### Taxonomic and functional profiling

For WGS sequences, taxonomic profiling was performed using MetaPhlAn3 (Metagenomic Phylogenetic Analysis v3.0) with the “—ignore_eukaryotes” flag to remove contamination.^51^ Prior to functional profiling by HUMAnN3 (HMP Unified Metabolic Analysis Network v3.0),^51^ contaminant host reads were removed *in silico* by aligning the sequences to the human genome (GRCh37/Hg19) using the KneadData integrated Bowtie2 (V.2.3.4.1) tool.^52^ Species and functional pathways with relative abundances > 0.01% and > 0.0001%, respectively, and prevalence > 5% were included in the further analyses.

We employed a standardized Mothur pipeline to process the 16S rRNA gene sequence data to profile up to the genus level. In summary, our pipeline aimed to adhere to established guidelines for processing Illumina sequencing data.^53^ All sequences underwent chimera screening using VSEARCH tool and were subsequently clustered into operational taxonomic units (OTUs) using the opticlust algorithm with a 3% distance threshold. Further “classify.otu” command was performed to determine the consensus taxonomic classification for the OTUs. All sequence processing was performed using Mothur software (version 1.48.0).

### Statistical analysis

All statistical analyses were carried out using R (version 4.0; https://www.R-project.org/) unless otherwise stated. We identified significantly differentially abundant taxonomic and functional features between the two groups in each dataset using LEfSe (Linear discriminant analysis (LDA) Effect Size) (https://huttenhower.sph.harvard.edu/lefse).^21^ LEfSe uses a non-parametric Kruskal-Wallis (KW) sum-rank test to identify significantly different features along with their respective effect sizes (LDA score). LDA score ≥ 2.0 and ≤ −2.0 with a *p-value* < 0.05, was considered significant. We also performed multivariate analyses in the presence of potential confounders. For multivariable analyses, we used the MaAsLin2 (Microbiome Multivariable Association with Linear Models v2.0) R package to fit general linear models to our data.^54^ MaAsLin2 was run first, in the absence of any covariate to compute “crude coefficients” and then in the presence of covariates (age, sex, comorbidity) to compute “adjusted coefficients”. Consequently, we used a linear regression model to analyze the relationship between MaAsLin2-derived crude and age-, sex-, comorbidity-adjusted coefficients to assess if the crude coefficients are meaningfully affected by the potential covariates. For the meta-analysis, we first converted the feature relative abundances into arcsine-square root-transformed proportions and used “escalc” function from the metafor R package to compute standardized mean difference (SMD) in terms of Cohen’s *d* .^55^ Consequently, random-effects model estimates were calculated from the SMD using the “rma.mv” function of the same package. Finally, meta-analysis results were visualized using the ggforestplot R package. P-values obtained from random effects were adjusted using the FDR method to adjust for multiple hypothesis testing. Any species or pathway present in all datasets with an effect size greater than 0.25 or less than −0.25 at FDR < 0.05 was considered reproducible and significantly differentially abundant across the datasets. Statistical significance was set at *p* <0.05. The degree of variability among studies in all meta-analyses was assessed by employing $I^{2}$ statistics and Cochran’s Q-test to determine the statistical significance of heterogeneity.^56^ We considered $I^{2}$ <60% as acceptable heterogeneity in our random-effect meta-analyses.

### Taxonomic and functional diversity and Firmicutes/Bacteroidetes ratio analysis

Alpha-and beta-diversity, gene richness, and Firmicutes/Bacteroidetes (F/B) ratio analyses were performed for each dataset. Prior to diversity analyses, for the sake of normalization, all the samples were rarefied to 90% of the lowest sample depth in a dataset specific way. Rarefaction and diversity analyses were performed with the phyloseq (v1.26.1) R package.^57^ We calculated three standard metrics of alpha diversity: Shannon’s and Simpson’s diversity indices for species and observed richness for gene families. Beta diversity was estimated using the Bray-Curtis dissimilarity metric. Statistical significance of the difference in alpha and beta diversity was tested independently between the two groups using the Wilcoxon rank-sum test. Next, we summarized the taxonomic profiles of the samples up to the phylum level and calculated the F/B ratio from the relative abundances of Firmicutes and Bacteroidetes phyla. The Wilcoxon rank-sum test was used to estimate the statistical significance of the F/B ratio between the groups. We also performed the same statistical analysis to assess the significance of the differences in gene richness between the control and obese groups.

### Random forest-based classifier

Machine learning analyses were performed using the MetAML (Metagenomic prediction Analysis based on Machine Learning) tool, which uses taxonomic and functional relative abundance as inputs. Random forest (RF) algorithm was used as a backend classifier in MetAML as it outperforms other machine learning algorithms with microbiome data^23.^ We performed three different modes of prediction, where we tested inside-dataset prediction capability (cross-validation, CV), across-dataset prediction performance (cross-study validation, CSV), and the leave-one-dataset-out (LODO) approach that mimics the meta-analysis. We assessed inside-dataset prediction accuracies by 10-fold CV, where a balanced proportion of the obese and control samples was used from the same dataset for both learning and validation in each fold. Each 10-fold cross-validation was iterated 20 times, and the average accuracy of the 200 validation folds was reported. Transportability of the model across datasets to evaluate generalizability and to determine whether the taxonomic and functional features are predictable across the datasets were assessed by performing CSV. In CSV, the model was trained on a dataset and validated on a completely independent dataset to evaluate reproducibility. The CSV was repeated for all combinations of independent datasets. Moreover, we assessed the predictability of the model when trained on multiple datasets using the LODO approach, also known as the hold-out study. In this setting, the model was learned from pooled samples from all datasets, except the one that was used for model validation. The test and validation datasets for this purpose were chosen independently. Thus, nine sets of LODO experiments, each for one dataset, were performed separately both for the species and pathway features. Furthermore, the specificity of the prediction is assessed by randomly shuffling the class labels of the samples and comparing the results with the original class. We used area under the receiver operating characteristic curve (AUC) value as a metric for the prediction performance. The potential discriminative features for classification were further obtained through internal feature ranking in the LODO study. Briefly, we first computed the minimum number of features required for classification by sequential addition of features, and subsequently estimated the AUC values. We ranked the minimum discriminative features capable of distinguishing samples for each LODO analysis. Subsequently, we computed the average rank for these features, that is, the obesity-associated signatures.

Replicates from the same country of origin were sought to investigate intra- and inter-country feature variability. Apart from China in the WGS, UK, and USA in the 16S rRNA-seq data, obtaining multiple datasets from the same region proved challenging. To address this limitation, we employed clustering based on overall dietary patterns from a previous study^34^ to group datasets from different countries. Following this strategy, we clustered the datasets into five distinct clusters: (1) Chinese cluster (CHN) including China:1 and China:2; (2) British cluster (BIN) including Great Britain (GBR), United Kingdom (UK), Ireland, and the Netherlands; (3) Scandinavian cluster (SCN) including Sweden and Denmark; (4) Israeli cluster (ISR); and (5) Kazakh cluster (KAZ). We performed LODO on these diet clusters using signature features to evaluate their classification performance. For the validation purpose, we used the France dataset as it affiliates to a distinct diet cluster,^34^ which is expected to comprise heterogeneities markedly divergent from other clusters.

### Identification of reproducible contributors of signature pathway shifts

To pinpoint the taxonomic contributors to the functional shifts, we used the FishTaco (*f*unct*i*onal *sh*ifts’ *ta*xonomic *co*ntributors) tool, a state-of-the-art computational framework that systematically integrates taxonomy with functional shifts to trace back to specific contributor taxa according to the protocol described by Manor and Borenstein^27^ with minor modifications. Briefly, we first manually curated all the genes and their respective copy numbers for all the species involved in our study from Integrated Microbial Genomes and Microbiomes (IMG) database version 5.0 (https://img.jgi.doe.gov/). We subsequently constructed the gene-to-pathway mapping file using the IMG and MetaCyc database version 23.1 (https://metacyc.org/) depending on the presence-absence pattern of a gene in a pathway. The pathway copy number was computed from the gene-to-pathway mapping file and the average gene copy number of the genes constituting the pathway. FishTaco exploits the pathway copy number (genomic content) of each taxon and the feature abundance information to identify the “Direct”- and “Indirect”-contributors of a functional shift. Control-enriched taxa encoding a control-associated function are “direct contributors” to this function. In contrast, obese-enriched taxa that lack that particular function (or present at a relatively low copy number) are termed as “Indirect contributors” of that function.^27^ Finally, FishTaco was implemented on each independent dataset, and the contributors to the signature functions obtained from the LODO experiments were identified. As LODO feature ranking retrieved the best discriminative features but did not reflect their class enrichment, we recovered their associated class from the meta-analysis result without considering any cutoff.

## Supplementary Material

Supplemental Material

S7.jpg

S1.jpg

S5.jpg

S9.jpg

S3.jpg

S4.jpg

S2.jpg

S8.jpg

Figure S10.jpg

S6.jpg

## Data Availability

Data sharing is not applicable to this study, as no new data were created. However, the authors confirm that the data supporting the findings of this study are available within the article, its supplementary materials, and referenced publications from which the data were extracted.

## Abbreviation

AUC, area under the receiver operating characteristic curve; BMI, body mass index; CSV, cross-study validation, CV, cross-validation; FDR, false discovery rate; F/B, Firmicutes/Bacteroidetes; LDA, linear discriminant analysis; LODO, leave-one-dataset-out; OTU, operational taxonomic unit; RF, random forest; ROC, receiver operator curves; SCFA, short-chain fatty acid, SMD, standardized mean difference; WGS, whole genome sequence.
