# Supplementary figures and images for: Meta-analysis reveals obesity associated gut microbial alteration patterns and reproducible contributors of functional shift
Source: Gut Microbes. 2024 Jan 24;16(1):2304900. doi: 10.1080/19490976.2024.2304900 (PMC10810176; doi:10.1080/19490976.2024.2304900)

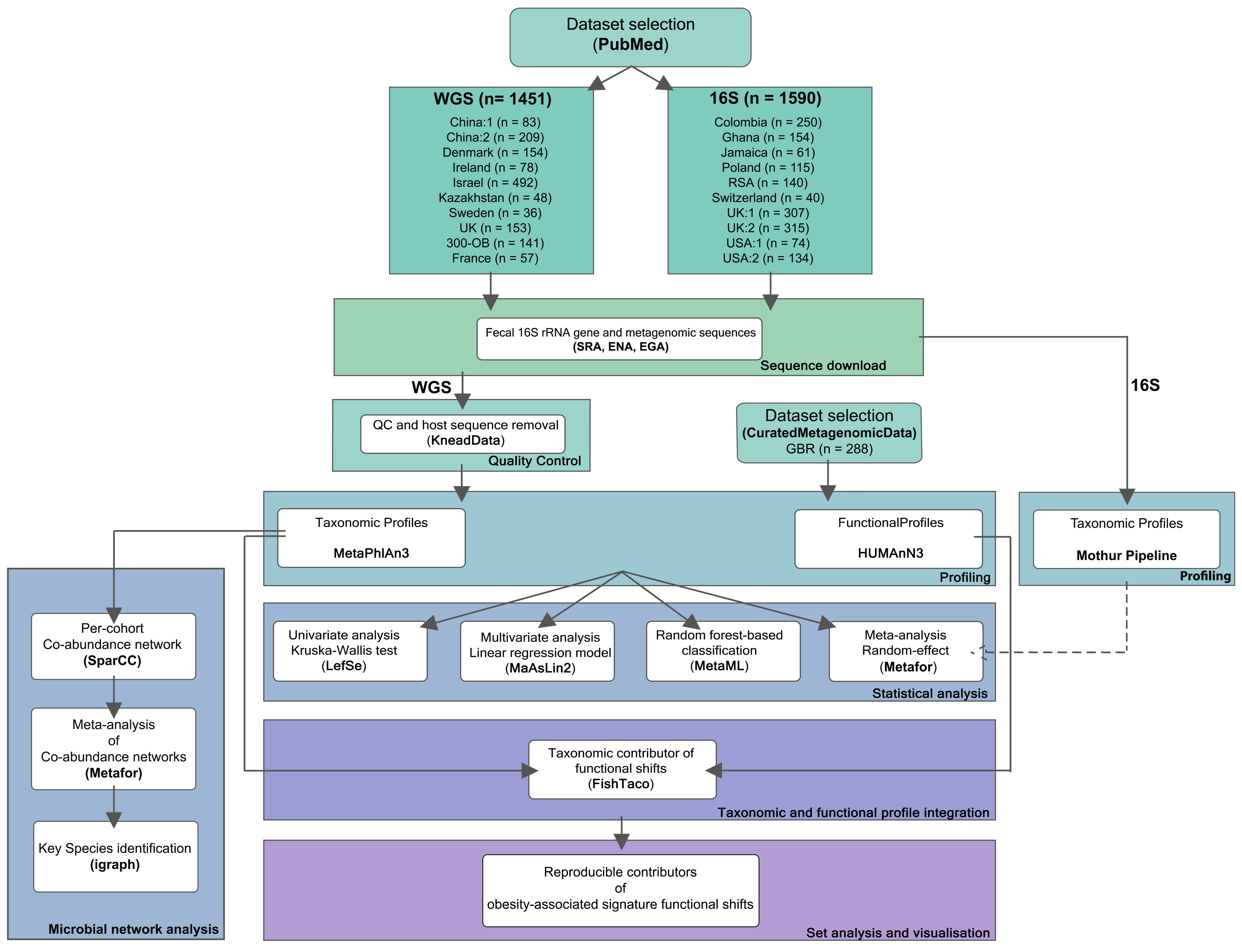

Supplement: S1.jpg [file KGMI_A_2304900_SM2490.jpg]

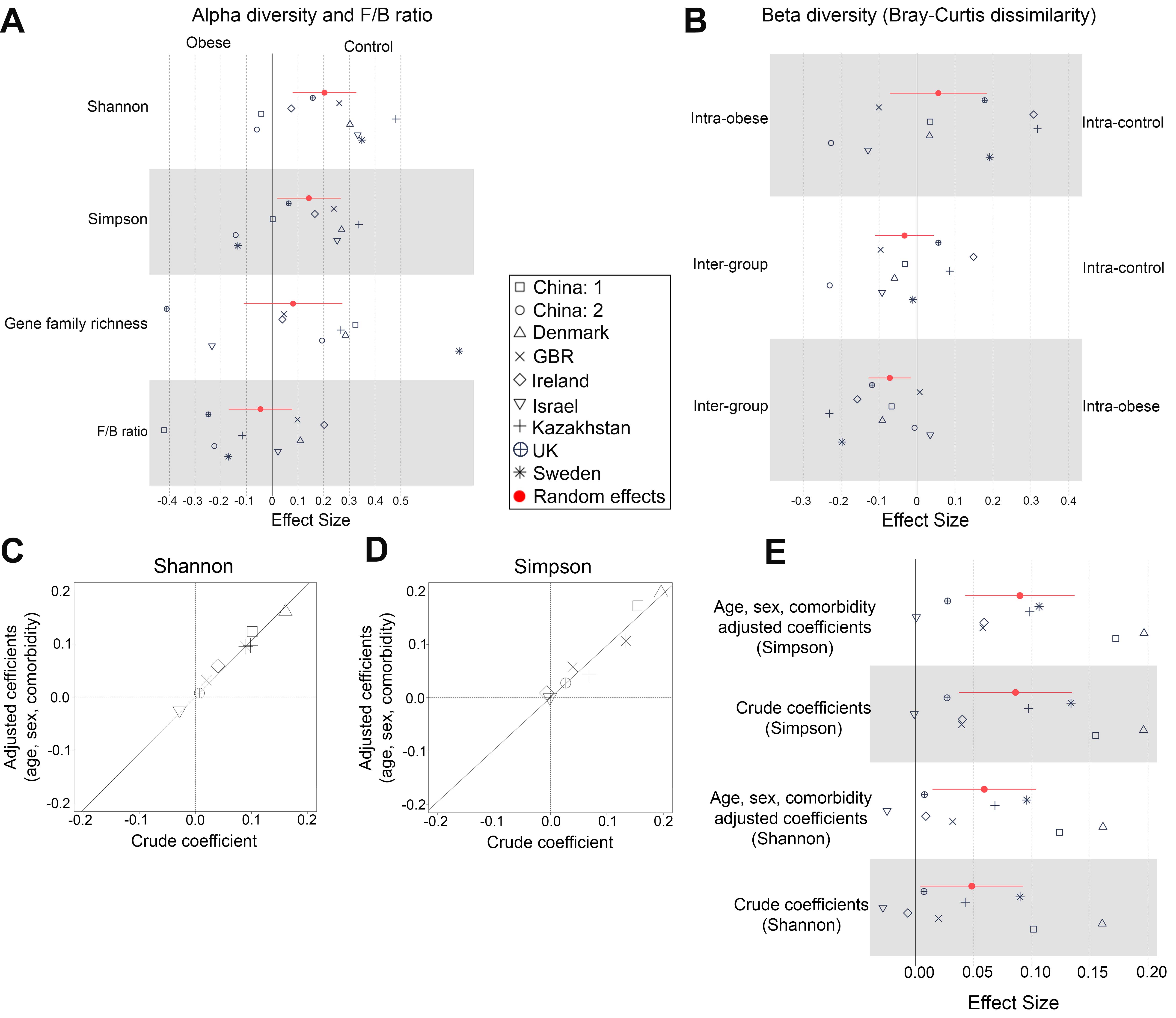

Supplement: S3.jpg [file KGMI_A_2304900_SM2487.jpg]

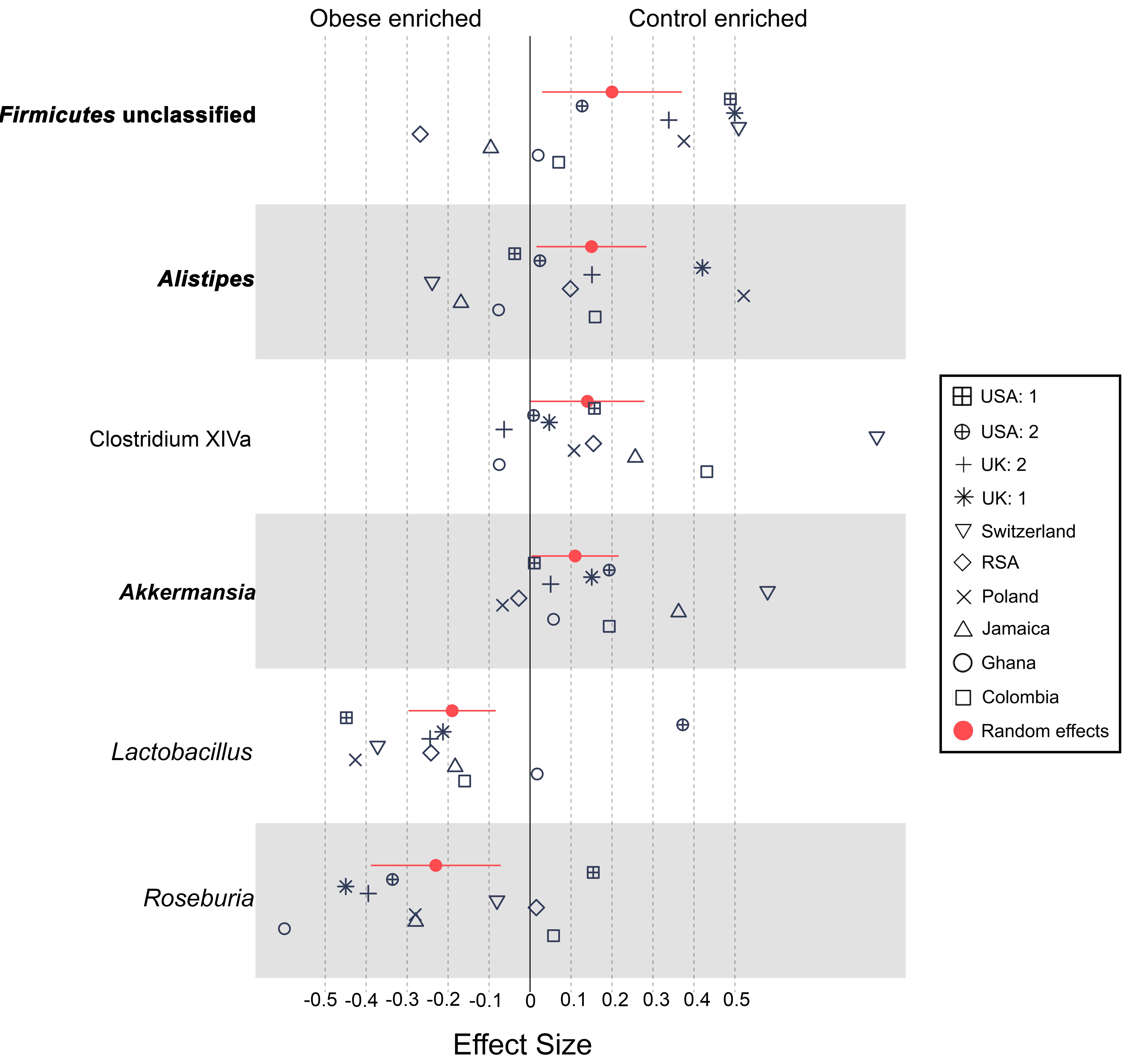

Supplement: S4.jpg [file KGMI_A_2304900_SM2486.jpg]

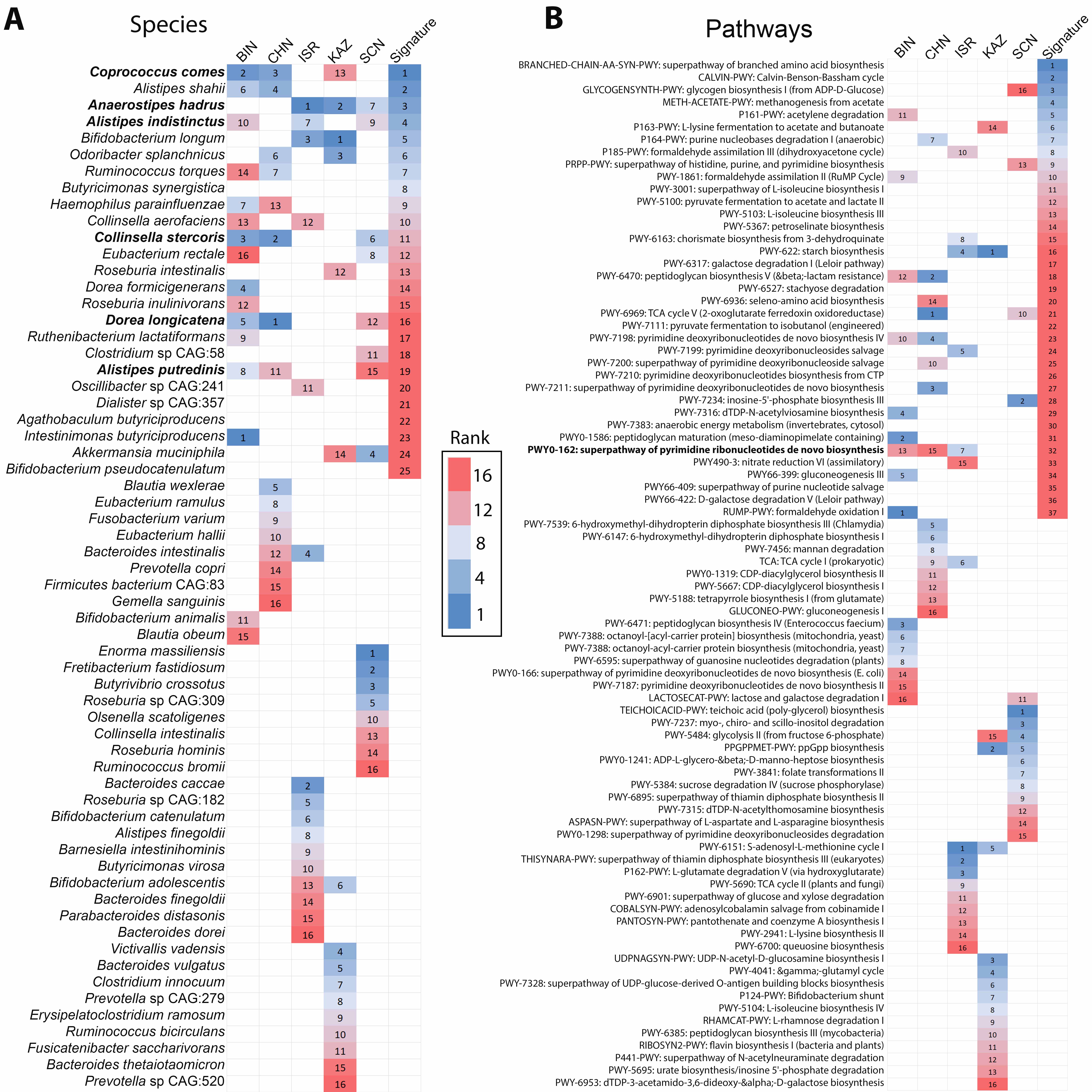

Supplement: Figure S10.jpg [file KGMI_A_2304900_SM2483.jpg]
